# Targeting senescent cells in translational medicine

**DOI:** 10.15252/emmm.201810234

**Published:** 2019-11-19

**Authors:** Marta Paez‐Ribes, Estela González‐Gualda, Gary J Doherty, Daniel Muñoz‐Espín

**Affiliations:** ^1^ Department of Oncology CRUK Cambridge Centre Early Detection Programme Hutchison/MRC Research Centre University of Cambridge Cambridge UK; ^2^ Department of Oncology Cambridge University Hospitals NHS Foundation Trust Cambridge Biomedical Campus Cambridge UK

**Keywords:** age‐related disorders, cellular senescence, SASP, senolytic drugs, senoprobes, Ageing, Pharmacology & Drug Discovery

## Abstract

Organismal ageing is a complex process driving progressive impairment of functionality and regenerative potential of tissues. Cellular senescence is a state of stable cell cycle arrest occurring in response to damage and stress and is considered a hallmark of ageing. Senescent cells accumulate in multiple organs during ageing, contribute to tissue dysfunction and give rise to pathological manifestations. Senescence is therefore a defining feature of a variety of human age‐related disorders, including cancer, and targeted elimination of these cells has recently emerged as a promising therapeutic approach to ameliorate tissue damage and promote repair and regeneration. In addition, *in vivo* identification of senescent cells has significant potential for early diagnosis of multiple pathologies. Here, we review existing senolytics, small molecules and drug delivery tools used in preclinical therapeutic strategies involving cellular senescence, as well as probes to trace senescent cells. We also review the clinical research landscape in senescence and discuss how identifying and targeting cellular senescence might positively affect pathological and ageing processes.

## Graphical abstract

This article reviews the broad topic of cellular senescence, how to manipulate it ‐ novel probes and nanocarriers have potential diagnostic and therapeutic values ‐ and details a number of senotherapies that entered clinical trials.

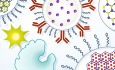

**Glossary**
Apoptosis: Controlled cell death that occurs in response to a variety of cellular stressors and as part of developmental programmes of multicellular organisms.
Autophagy: Regulated mechanism used by cells to maintain homeostasis and normal functioning by degradation of unnecessary or dysfunctional components.
Cellular senescence: Cellular state characterized by a stable cell cycle arrest and the implementation of a complex secretory phenotype (SASP) in response to different sources of damage and/or stress.
Cytokines: Broad range of small secreted proteins that have specific effects on the interactions and communications between cells and the immune system.
DDR (DNA Damage Response): Network of cellular pathways that sense, signal and repair DNA lesions. These include a set of DNA repair mechanisms, damage tolerance processes and cell cycle checkpoint pathways.
Metabolic reprogramming: Molecular adjustments in metabolic pathways that alter the bioenergetic profile and metabolism of the cell.
Nanoparticles: Particles between 1 and 100 nm in size with very large surface area to volume ratios that can be functionalized for specific drug delivery targeting.
Neutropenia: The abnormally low concentration of neutrophils in the blood. If severe, it can significantly increase the risk of infection.
OIS (Oncogene‐Induced Senescence): Robust and sustained antiproliferative response that can be induced by aberrant signalling resulting from an activating mutation of an oncogene, or the inactivation of a tumour suppressor gene.
Osteoclastogenesis: The development of osteoclasts (cells responsible for bone break down and resorption) from blood cells, specifically from monocytes/macrophages.
Oxidative stress: Imbalance caused by a higher production of free radicals that cannot be neutralized by the antioxidants produced inside the cell, resulting in damaged components.
Progeroid (mouse) model: Genetically engineered mouse model presenting a pronounced premature ageing and typical age‐related pathologies. In particular, the text refers to *Ercc1* ^−/Δ^ mice. Ercc1 is a DNA excision repair protein involved in genome maintenance.
Proteotoxic stress: Molecular response triggered by the accumulation of misfolded proteins within the cell, which may impair cellular function.
Renal glomerulosclerosis: Condition that refers to the scarring or hardening of the glomeruli in the kidney (the renal functional units). If left untreated, it can lead to kidney failure.
Replicative senescence: State of cellular senescence induced by the attrition of telomeres in the cells, which triggers a specific DNA damage response.
SAβgal (Senescence‐associated β‐galactosidase): Lysosomal enzymatic activity increased in senescent cells that catalyses the hydrolysis of β‐galactoside into monosaccharides. It is commonly used as a marker of cellular senescence.
SAHF (Senescence‐associated heterochromatin foci): Regions of facultative heterochromatin within the nucleus that allow the silencing of proliferation‐related genes in the cell. It is considered a common feature of cellular senescence.
Sarcopenia: Degenerative loss of skeletal muscle mass, quality and strength associated with ageing.
SASP (Senescence‐associated secretory phenotype): A robust secretion of molecules such as growth factors, chemokines, cytokines and extracellular matrix metalloproteases that occurs when a cell undergoes senescence.
Secretome: The set of all molecules and factors secreted by a cell into the extracellular space.
Senolytic drugs: Chemical compounds that selectively target and induce the death of senescent cells.
Senoprobes: Molecules that have been developed and engineered to analyse or detect senescent cells for diagnostic or experimental purposes.
Synovium: Soft connective tissue that lines the inner surface of spaces of diarthrodial joints, tendon sheaths and bursae.
Theranostic tools: Tools aimed at simultaneously detecting and eradicating a pathological lesion or damaged area of tissue.
Thrombocytopenia: Condition associated with low blood platelet counts.
TIS (Therapy‐Induced Senescence): A subtype of cellular senescence triggered by a therapeutic treatment such as chemotherapy or radiotherapy.

## Introduction

Severe or irreparable cellular damage triggers a stereotyped response across vertebrates based on a stable cell cycle arrest known as cellular senescence. Senescent cells can implement a complex paracrine response, including cytokines, chemokines, growth factors, proteases and extracellular matrix remodelling factors. This senescence‐associated secretory phenotype (SASP) can play differing roles depending on the physiological context (Muñoz‐Espín & Serrano, 2014; Pérez‐Mancera *et al*, 2014). A major consequence of senescence appears to prevent propagation of pre‐malignant cells, providing a crucial barrier to tumorigenesis (Collado & Serrano, 2010). Cell senescence participates in other physiological processes by promoting tissue repair and regeneration (Demaria *et al*, 2014; Yun *et al*, 2015) and plays a programmed role in morphogenesis during normal embryonic development in a damage‐independent fashion (Muñoz‐Espín *et al*, 2013; Storer *et al*, 2013). Senescent cells can elicit a tissue remodelling process that includes their own elimination, recruitment of phagocytic immune cells and mobilization of nearby progenitor cells. However, upon persistent damage or during ageing, senescent cell clearance is compromised and dysfunctional cells accumulate, contributing to the generation of a chronic pro‐inflammatory microenvironment that results in a diverse range of pathological manifestations.

**Figure 1:**
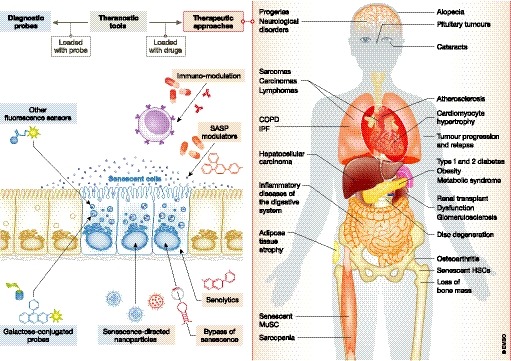
Therapeutic and diagnostic opportunities in senescence‐related disorders and during ageing Cellular senescence is associated with multiple human disorders, offering potential interventions for targeted therapeutic and diagnostic approaches. The development of galactose‐conjugated and fluorescent probes to detect and highlight senescent cells offers an important opportunity for longitudinal monitoring of senescence in clinical trials. Pharmacologically active small compounds known as senolytics inhibit pro‐survival pathways in senescent cells leading to apoptosis, a therapeutic strategy that may additionally be enhanced by the use of immune modulators promoting natural clearance of senescent cells. Also, a variety of drugs can manipulate the SASP and its detrimental effects, thus suggesting a potential clinical use. Interventions to bypass senescence represent an interesting alternative, although this approach should be taken with caution due to the risk of uncontrolled proliferation and cancer initiation. Finally, nanoparticles encapsulating cytotoxic drugs, tracers and/or small molecules can be used as theranostic tools, both for therapeutic and diagnostic purposes. Of note, the benefits of therapeutic approaches for the prevention or elimination of senescent cells *in vivo* have been validated in an increasing number of conditions. Genetic manipulation to inactivate the senescence pathway or to ablate senescent cells in murine models produced (mostly) a beneficial impact irrespective of the disorder or condition investigated, including adipose atrophy, cataracts, IPF, sarcopenia, kidney dysfunction, atherosclerosis, premature ageing of the haematopoietic system, osteoarthritis, cardiomyocyte hypertrophy, loss of bone mass, type 2 diabetes, tumorigenesis, neurological disorders and natural ageing. Furthermore, clearance of senescent cells by treatment with senolytic drugs, a more clinically relevant approach, showed *in vivo* benefits in, among other disorders, atherosclerosis, premature ageing of the haematopoietic system, myocardial infarction, IPF, osteoarthritis, osteoporosis, type 1 diabetes, obesity‐induced metabolic syndrome and neuropsychiatric disorders, tau‐dependent pathologies, cancer and natural ageing. IPF, idiopathic pulmonary fibrosis; HSC, hematopoietic stem cells; MuSC, muscle stem cells.

Cell senescence contributes to a wide variety of human age‐related pathologies, including cancer, fibrosis, cardiovascular diseases, obesity, type 2 diabetes, sarcopenia, osteoarthritis and neurological disorders (van Deursen, 2014; Muñoz‐Espín & Serrano, 2014). The genetic ablation of p16^ink4a+^ senescent cells in progeroid and chronic disease mouse models attenuates or even reverts tissue dysfunction, leading to an increased animal healthspan. p16^ink4a+^ senescent cell clearance can ameliorate adipose atrophy, sarcopenia, cataracts, cardiomyocyte hypertrophy, renal glomerulosclerosis, tumorigenesis, cancer progression, atherosclerosis, osteoarthritis and tau‐dependent disease (Baker *et al*, 2011, 2016; Childs *et al*, 2016; Demaria *et al*, 2017; Jeon *et al*, 2017; Bussian *et al*, 2018). Importantly, such clearance also increases mouse lifespan (Baker *et al*, 2016). For years, pharmacological clearance of senescent cells has proved very challenging. However, in the last few years, renewed research efforts have led to the development of senolytics, a collection of molecules capable of preferentially removing senescent cells (Childs *et al*, 2017; Soto‐Gamez & Demaria, 2017; Ovadya & Krizhanovsky, 2018). Taken together, emerging data suggest that cell senescence is causative of multiple human disorders (Fig 1), which can be induced by various stimuli including drugs used clinically, and that senolytics may provide exciting opportunities for therapeutic intervention.

**Figure 2:**
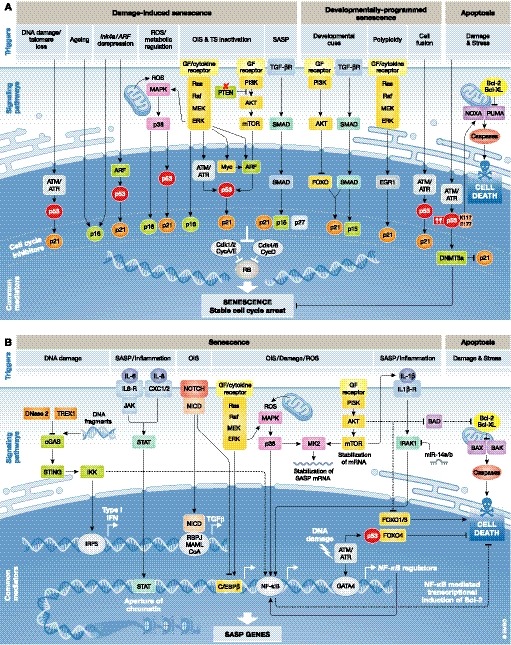
Regulation of the cell cycle arrest and inflammatory SASP in the induction of cellular senescence and its interconnection with apoptosis (A) Most senescence‐inducing triggers converge in the activation of the cell cycle inhibitor pathways p53/p21 and/or p16^INK^ ^4a^. These result in the inhibition of cyclin‐dependent kinase 1 (CDK1), CDK2, CDK4 and CDK6, which prevents the phosphorylation of the retinoblastoma protein (RB), leading to the suppression of S‐phase genes and an ensuing stable cell cycle arrest. DNA‐damaging triggers activate the DNA damage response (DDR) pathway resulting in the activation of p53 and p21. Ageing and epigenetic derepression of the Ink4a/ARF locus also lead to the activation of cell cycle inhibitors p16 and p21. ROS lead to the activation of the MAPK signalling pathway and its downstream effector p38. The aberrant expression of oncogenes or the loss of tumour suppressors leads to p53 activation through the Ras‐Raf‐MEK‐ERK or AKT signalling pathways, and TGFβ, and important factor of the SASP, leads to p15, p21 and p27 upregulation via SMAD signalling. Other triggers such as developmental cues and polyploidy activate the AKT, SMAD and/or Ras‐Raf‐MEK‐ERK pathway for p21 upregulation, while processes such as cell fusion signal through the DDR for p53 activation. In response to damage and different types of stress high levels of p53 with specific post‐translational modifications (such as acetylated K117 and E177) target DNMT3a, a suppressor of p21 and senescence, and trigger the apoptotic programme by upregulating PUMA and NOXA, which in turn activate the caspase cascade leading to cell death. (B) SASP implementation is orchestrated by the activation of the transcription factors NF‐κB and C/EBPβ through upstream signalling pathways. DNA‐damaging agents, ROS and OIS, generally activate the expression of SASP TFs via the AKT and/or the Ras‐Raf‐MEK‐ERK axis. In addition, DNA fragments are also known to trigger the activation of the cGAS/STING signalling, resulting in the activation of the IRF3 TF and subsequent transcription of Type 1 IFN. OIS‐derived SASP is dynamic and can also be orchestrated by NOTCH signalling, a process that restrains the inflammatory secretion by inhibiting C/EBPβ at initial stages, and allows the activation of SASP‐related super enhancers through NF‐κB later on. Accumulating increased levels of TFs reinforce the senescent phenotype through autocrine and paracrine signalling. SASP‐derived inflammatory chemokines such as IL‐6 and IL‐8 promote epigenetic modifications reinforcing the cell cycle arrest through the JAK/STAT cascade, while IL‐1α stimulates the activity of NF‐κB and C/EBPβ promoting a positive feedback loop on the secretion of other cytokines. Finally, senescence promotes survival networks by the regulation anti‐apoptotic pathways. This includes PI3K‐AKT signalling, which can inhibit pro‐apoptotic BAD and FOXO1/3, and phosphorylate caspase‐9; anti‐apoptotic FOXO4, that is present in senescent cells and interacts with p53; and NF‐κB, that may also promote survival responses by transcriptional induction of anti‐apoptotic proteins of the Bcl‐2 family. ATM/ATR, ataxia‐telangiectasia mutated and Rad3‐related homologue; IFN, interferon; OIS, oncogene‐induced senescence; ROS, reactive oxygen species; SASP, senescence‐associated secretory phenotype; TFs, transcription factors; TS, tumour suppressor.

Besides stable cell cycle arrest and SASP production (see Fig 2 for relevant signalling pathways), another hallmark of senescent cells is their resistance to damage‐induced apoptosis through survival pathway upregulation (Childs *et al*, 2014, 2017; Soto‐Gamez *et al*, 2019). Some senolytics exploit their capacity to stimulate cell death pathways (see below). In the absence of a single universal hallmark, senescent cells can be identified by combining a number of markers (Sharpless & Sherr, 2015), including upregulation of *p16* ^*ink4a*^ and other cell cycle inhibitors, exclusion of proliferative markers, formation of specialized heterochromatin domains (senescence‐associated heterochromatin foci, SAHF) and persistent activation of the DNA damage response (DDR) machinery. Although imperfect, detection of increased activity of lysosomal senescence‐associated β‐galactosidase (SAβgal) remains the most widely used indicator of cellular senescence (Sharpless & Sherr, 2015), explaining why many senescence detection probes are based on detecting its enzymatic activity.

Here, we review approaches to identify and therapeutically target senescent cells (Fig 1). These strategies comprise senolytics and novel drug delivery tools to target senescence. Additional innovative interventions to manipulate cell senescence include the modulation of the SASP and associated signalling pathways, immunotherapy and promoting the artificial reactivation of proliferation. We discuss novel probes for senescent cell visualization, and their potential utility in medical diagnosis as well as for monitoring accumulation or elimination of senescent cells. We comment on the clinical development of senescence‐targeted strategies and future translational considerations, highlighting novel opportunities as well as current challenges.

## Therapeutic approaches

### Senescent cells as target of therapy

**Figure 3:**
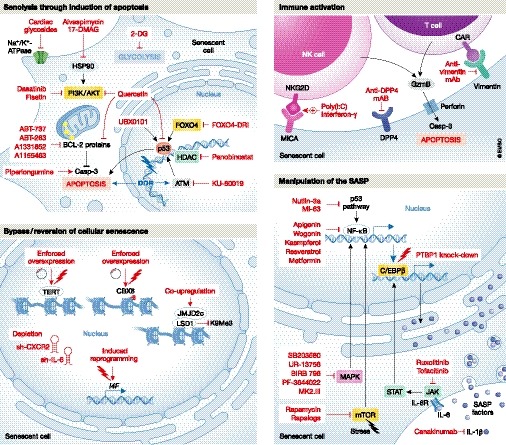
Therapeutic approaches targeting cellular senescence To prevent the deleterious effects of cellular senescence, four different strategies can be potentially implemented. The inhibition of pro‐survival pathways by the use of apoptosis‐inducing drugs is a leading approach. First and second generation of inhibitors of the BCL‐2 cell death regulator family of proteins can induce selective apoptosis of senescent cells. Targeting senescence metabolism through glycolysis blockade and attenuation of ATM, HDAC, FOXO4 activities as well as the PI3K cascade have also been reported as effective approaches. A second strategy is the activation of the immune system against senescent cells to stimulate their clearance. Enhancing the cytotoxic activity of NK against senescent cells, and manipulating the humoral innate immunity with the use of antibodies against receptors, such as DPP4 and vimentin, are proposed attractive strategies. Thirdly, manipulation of the SASP without compromising the cell cycle arrest of senescent cells has also proven beneficial in particular settings. A large number of molecules can interfere with NF‐κB and C/EBPβ transcriptional activities or their upstream regulators, dampening the expression of SASP factors, such as IL‐1, IL‐6 and IL‐8, and thus reducing the senescence‐derived inflammatory milieu. Lastly, genetic and epigenetic manipulation of cells, including the induction of reprogramming, have been proposed as a means of bypassing or reverting the state of cellular senescence, although these approaches should be taken with caution given the potential risk of cancer initiation. BCL‐2, B‐cell lymphoma 2; CAR, chimeric antigen receptor; GzmB, granzyme B; HDAC, histone deacetylase; HSP90, heat shock protein 90; i4F, inducible four Yamanaka factors; LSD1, lysine‐specific histone demethylase 1A; MICA, MHC class I polypeptide‐related sequence A; NK, natural killer; TERT, telomerase reverse transcriptase.

Senescence and apoptosis have been proposed as alternative cell fates in the context of damage and stress. Pro‐apoptotic cellular changes are often actively anti‐senescent (Fig 2A), while senescent cells are highly resistant to apoptosis. Several molecular mechanisms of the senescent phenotype directly contribute to increased survival (Fig 2B) (Childs *et al*, 2014; Soto‐Gamez *et al*, 2019). Accordingly, targeting pro‐survival pathways, i.e. those involving the BCL‐2 family of proteins, the p53 or PI3K/AKT pathways (Table EV1, Figs 2B and 3), is currently the most common strategy to promote senescent cell elimination in damaged or aged tissues.

#### Inhibition of pro‐survival pathways by single senolytics

Deregulation of the BCL‐2 family of proteins, major regulators of programmed cell death, is commonly observed in multiple haematological, autoimmune, degenerative disorders and cancer (Singh *et al*, 2019). Navitoclax (ABT‐263), a specific inhibitor of BCL‐2, BCL‐xL and BCL‐W, induced selective apoptosis of a variety of cells undergoing senescence induced by ionizing radiation, oncogene expression or replicative exhaustion, including human umbilical vein epithelial cells (HUVECs), human lung fibroblasts and MEFs, but not in senescent human primary preadipocytes (Zhu *et al*, 2016). Administration of ABT‐263 also depleted senescent cells *in vivo*, mitigating premature ageing of the haematopoietic system in sublethally irradiated mice, thereby participating in bone marrow and muscle haematopoietic stem cells rejuvenation in normally aged mice (Chang *et al*, 2016). In addition, ABT‐263 treatment reduced the expression levels of several SASP factors in aged murine lungs, including *Cdkn2a* (encoding for p16^ink4a^), *Tnfa* (encoding for TNF‐α) and *Ccl5* (encoding for CCL5). In a model of articular joint injury, the selective elimination of senescent cells in the articular cartilage and synovium by ABT‐263 reduced features of post‐traumatic osteoarthritis (Jeon *et al*, 2017); age‐related symptoms were ameliorated by targeting senescent osteoblast progenitors obtained from aged mice (Kim *et al*, 2017a). ABT‐263 reduced the levels of several SASP components by eliminating the senescent progenitor cells, resulting in attenuation of osteoclastogenesis in bone marrow stromal cell cultures from aged mice (Kim *et al*, 2017a).

ABT‐737 is an analogue of ABT‐263, validated in IMR90 human fibroblasts and in mouse embryonic fibroblasts (MEFs) where the anti‐apoptotic proteins BCL‐2, BCL‐W and BCL‐xL are highly induced upon different senescence‐inducing stimuli (Yosef *et al*, 2016). ABT‐737 eliminated senescent cells in the lungs and epidermis of irradiated mice, resulting in increased hair‐follicle stem cell proliferation. However, both ABT‐263 and ABT‐737 are toxic for neutrophils and platelets (Cang *et al*, 2015), which may limit their clinical development. Second‐generation inhibitors of the BCL‐2 family of proteins include the BCL‐xL inhibitors A1331852 and A1155463, which induced selective apoptosis of senescent HUVEC and IMR90 cells, but not of senescent human preadipocytes (Zhu *et al*, 2017). Preadipocytes appear resistant to BCL2 family member blockade, suggesting heterogeneity in cell‐intrinsic senescence pathways (Zhu *et al*, 2016). These BCL‐xL selective inhibitors enhance the efficacy of standard chemotherapy in mouse models of breast cancer, non‐small‐cell lung cancer (NSCLC) and ovarian cancer, while avoiding the exacerbation of cytotoxic chemotherapy‐induced neutropenia observed with ABT‐263 (Leverson *et al*, 2015). While more BCL‐2 family inhibitors are currently in development, targeting the BH4 domain is promising for enhancing senolytic sensitivity; BH4 domain is present in all the pro‐survival members of the family (BCL‐2, BCL‐xL, BCL‐W, MCL‐1 and BFL‐1) and necessary for their anti‐apoptotic activity (Liu *et al*, 2016).

Piperlongumine, isolated from trees of the genus *Piper*, is a natural senolytic agent. Initially found to inhibit tumour growth in a xenograft mouse model of NSCLC, it was later shown to trigger apoptosis and preferentially kill senescent cells induced by oncogene expression, ionizing radiation or replicative exhaustion (Wang *et al*, 2016). Panobinostat (an FDA‐approved histone deacetylase inhibitor) has senolytic activity in NSCLC, and head and neck squamous cell carcinoma (HNSCC) cell lines previously treated with clinically relevant cytotoxic drugs (cisplatin and paclitaxel) (Samaraweera *et al*, 2017). Panobinostat increased caspase 3/7 activity and decreased Bcl‐xL expression in chemotherapy‐induced senescent cells. Recently, cardiac glycosides (CGs) have been identified as a family of compounds with potent senolytic activity (Guerrero *et al*, 2019; Triana‐Martínez *et al*, 2019). CGs target the cell membrane Na^+^/K^+^‐ATPase pumps causing a disbalanced electrochemical gradient that makes senescent cells more vulnerable. These compounds have been validated *ex vivo* in senescent preneoplastic cells and *in vivo* in models of lung fibrosis, therapy‐induced senescence, and aged mice.

#### Combinations of drugs with senolytic activity

The tyrosine kinase inhibitor dasatinib (which inhibits SRC, c‐KIT, ephrin receptors and other kinases) and the flavonoid quercetin (which has multiple targets including kinases and receptors, and inhibits the PI3K‐AKT pathway) are effective in combination at eliminating senescent cells *in vitro* and *in vivo*, selectively targeting a wide range of senescent cell types (Zhu *et al*, 2015; Roos *et al*, 2016). A single administration of dasatinib and quercetin in aged mice was sufficient to improve cardiovascular function and also reduced the expression of p16^ink4a^ and prevalence of SAβGal‐positive cells after localized limb irradiation (Zhu *et al*, 2015). Periodic drug administration extended healthspan in progeroid mice, delaying age‐related symptoms and pathologies. The combination of dasatinib and quercetin (D + Q) in mice resulted in increased survival and improved health. D + Q treatment prevented and alleviated physical dysfunction in naturally aged mice and mice transplanted with senescent preadipocytes, and reduced senescent cell prevalence and pro‐inflammatory cytokine secretion in explants of human adipose tissue obtained from obese individuals (Xu *et al*, 2018).

It is thought that cell senescence contributes to idiopathic pulmonary fibrosis (IPF), a progressive and debilitating chronic disease with limited therapeutic options (Naikawadi *et al*, 2016). *Ex vivo* treatment of mouse primary alveolar epithelial type II cells from fibrotic lungs with D + Q reduced senescence and fibrosis markers (Lehmann *et al*, 2017). The combination mitigated fibrotic lung disease in a bleomycin‐injury mouse model, clearing senescent cells and improving pulmonary and physical health of treated animals (Schafer *et al*, 2017). D + Q also cleared senescent cells in the medial segments of blood vessels, improving the vascular phenotype associated with age‐related vascular pathology (Roos *et al*, 2016). Likewise, chronic treatment reduced intimal aortic plaque calcification in a hypercholesterolaemic mouse model of atherosclerosis. D + Q are well‐tolerated drugs and are now being trialled in human patients in a variety of senescence‐associated conditions (see Targeting senescence clinically in age‐related disorders).

Of note, a panel of flavonoid polyphenols distinct from quercetin has been screened for senolytic activity. Fisetin reduced senescence markers in multiple tissues in progeroid and naturally aged mice (Yousefzadeh *et al*, 2018), and administration of fisetin in normally aged mice restored tissue homeostasis, reduced age‐related dysfunction and extended lifespan. Future therapeutic outcomes will be required for initial proof of principle of combination therapies.

#### Targeting pathways involved in senescence

The p53 axis, another key controller of apoptosis and senescence, is a promising target for novel senolytic strategies. FOXO transcription factors can interact with p53, inhibiting p53‐mediated apoptosis and favouring cell cycle arrest and senescence (Wang *et al*, 2008). A D‐retro inverso (DRI)‐isoform of FOXO4 was developed which causes p53 nuclear exclusion and resultant death of senescent cells (Baar *et al*, 2017). FOXO4‐DRI selectively eliminated human senescent fibroblasts *in vitro* through p53‐mediated apoptosis. In naturally and experimentally aged mice, FOXO‐DRI counteracted doxorubicin‐induced senescence and chemotoxicity, minimizing hepatotoxicity and loss of body weight, and reducing features of frailty and loss of renal function (Baar *et al*, 2017).

High‐throughput experimental approaches are capable of identifying novel senolytic drugs and targets. A screening platform based on SAβgal activity identified HSP90, a ubiquitously expressed chaperone with a role in protein stabilization, as a potential target (Fuhrmann‐Stroissnigg *et al*, 2017). HSP90 inhibitors downregulate the anti‐apoptotic PI3K/AKT pathway and reduce senescence markers in a variety of human and mouse cell lines. In a progeroid mouse model, the HSP90 inhibitor 17‐DMAG [alvespimycin, which has been tested in clinical trials in different solid tumours and lymphomas (Trepel *et al*, 2010)] reduced p16^ink4a^ expression levels, extended healthspan and delayed the onset of various age‐related clinical markers (Fuhrmann‐Stroissnigg *et al*, 2017). A similar high‐throughput screening approach identified KU‐60019, an inhibitor of the DDR protein ataxia‐telangiectasia mutated (ATM), as an effective anti‐senescent agent (Kang *et al*, 2017). ATM can mediate mechanisms that control senescence through regulating lysosomal acidification. Treatment with KU‐60019 decreased SAβgal activity in senescent fibroblasts, removed dysfunctional mitochondria and resulted in metabolic reprogramming. KU‐60019 therapy accelerated cutaneous wound healing in aged mice, and inhibition of ATM activity also attenuated senescence (Kang *et al*, 2017); KU‐60019 is therefore a promising candidate target for treatment of age‐related diseases.

Senescent cells are “hypermetabolic”, and this may potentially be therapeutically targetable (Dörr *et al*, 2013). Metabolic reprogramming is required for senescent cells to cope with the high energetic demands of the senescent programme, including SASP‐coupled proteotoxic stress (featured by high production of SASP factors, increased oxidative stress resulting in misfolded or toxic proteins, and increased endoplasmic reticulum stress/unfolded protein response/ubiquitination/autophagy cascade). Accordingly, senescent cells are more sensitive to treatment with 2‐DG, a decoy substrate for glycolytic metabolism or specific inhibitors of lysosomal V‐ATPases (Dörr *et al*, 2013). How metabolically targeted drugs can achieve sufficient specificity for senescent over non‐senescent cells *in vivo* to allow successful translation remains an open question.

### Manipulation of the SASP

Although inducing the selective apoptosis of senescent cells using senolytic drugs could be therapeutically beneficial, dampening the detrimental effects of the SASP without compromising senescent cell cycle arrest may prove more advantageous in particular settings. Seminal work showing that the SASP and cell cycle arrest are independently regulated (Coppé *et al*, 2011) opened the door to differentially targeted therapeutic approaches. Senescent cells implement programmed secretion of growth factors, matrix metalloproteases, chemokines and cytokines that can trigger a wide range of autocrine and paracrine effects. Some of these [such as immune activation and reinforcement of growth arrest and differentiation (Hong *et al*, 2007; Anestakis *et al*, 2015)] are crucial for the resolution of tissue damage, but others (such as cell growth, migration and invasion) can be disadvantageous in certain contexts including cancer (Pérez‐Mancera *et al*, 2014; Gonzalez‐Meljem *et al*, 2018; Lee & Schmitt, 2019). Activation of various signalling pathways, including key drivers such as mammalian target of rapamycin (mTOR), mitogen‐activated protein kinase (MAPK) signalling, phosphoinositide 3 kinase (PI3K) signalling and GATA4/p62‐mediated autophagy, orchestrates this complex secretome (reviewed in Faget *et al*, 2019) (Fig 2B). These cascades converge in the activation of the NF‐κB and the CCAAT/enhancer binding protein beta (C/EBPβ) pathways. The great diversity of potential targets able to modulate the cascades driving the expression of the SASP prompted the development of a number of molecules and antibodies to interfere with NF‐κB and C/EBPβ transcriptional activities at different levels (Table EV2 and Fig 3).

#### Modulation of the upstream regulators of NF‐κB activity

mTOR is a serine/threonine kinase implicated in a wide variety of cellular processes. It is thought to interact with the MAPK pathway by increasing the translation of MAPKAPK2 (Herranz *et al*, 2015), ultimately resulting in NF‐κB activation and nuclear translocation. mTOR can also regulate membrane‐bound IL‐1α expression (Laberge *et al*, 2015), rendering it an attractive target for selective inhibitors. mTOR inhibition by rapamycin in normal human fibroblasts and non‐tumorigenic human breast cells suppresses the secretion of inflammatory cytokines including IL‐6, and selectively decreases IL‐1α translation, thereby diminishing NF‐κB transcriptional activity (Laberge *et al*, 2015). Rapamycin also suppresses the ability of senescent fibroblasts to stimulate prostate tumour growth in mice (Imrali *et al*, 2016) and blocks the translation of MAPKAPK2, leading to degradation of several SASP components transcripts, including IL‐8 and IL‐1α (Herranz *et al*, 2015). Treatment of murine lung WI‐38 fibroblasts with rapamycin resulted in a significant decrease in IL‐6, IL‐1β and Vcam‐1 transcription and decreased Stat3 pathway activation (Wang *et al*, 2017). Newer mTOR inhibitors with comparatively advantageous pharmacological properties have been developed and may also target detrimental effects of the SASP (Lamming *et al*, 2013; Leontieva *et al*, 2015). The effects of such “rapalogs”, including everolimus, temsirolimus and deforolimus on senescence remain however unclear.

Inhibitors of members of the MAPK pathway have also been investigated as SASP modulators. The p38MAPK inhibitor SB203580 reduces mRNA levels and secretion of several SASP components reducing NF‐κB transcriptional activation and paracrine effects of the SASP in human senescent cells (Freund *et al*, 2011). The next‐generation p38MAPK inhibitors UR‐13756 and BIRB 796 also suppress IL‐6 expression in human senescent fibroblasts, and treatment of cells with the MAPKAPK2 inhibitors PF‐3644022 and MK2.III attenuates the SASP (Alimbetov *et al*, 2016). In addition, treatment of astrocytes with ginsenoside F1, an enzymatically modified derivative of ginsenoside Rg1 that targets p38MAPK, robustly decreased IL‐6 and IL‐8 secretion (Hou *et al*, 2018). Conditioned media from senescent astrocytes treated with ginsenoside F1 were less able than controls to induce paracrine‐activated cell migration of glioblastoma cells.

Nutlin‐3a results in p53 stabilization by inhibiting Mdm2 and was shown to inhibit the activity of NF‐κB in a p53‐dependent manner (Dey *et al*, 2008). Nutlin‐3a is therefore an attractive anti‐cancer therapy since it could simultaneously activate p53 and suppress NF‐κB. Indeed, the cytokine response to DDR requires ATM, NBS1 and CHK2, but not the cell cycle arrest enforcers p53 and pRb (Rodier *et al*, 2009). Nutlin‐3a treatment of human fibroblasts not only decreased the secretion of certain SASP interleukins, but the collected conditioned medium was able to suppress breast cancer cell invasiveness (Wiley *et al*, 2018). MI‐63, a next‐generation Mdm2 inhibitor, showed similar results (Wiley *et al*, 2018), confirming the potential of these drugs to attenuate detrimental paracrine effects of the SASP. Many Mdm2‐MdmX inhibitors have entered clinical trials with the hope of restoring p53 function in a variety of malignancies; thus far, no drugs in this class have been approved.

#### Direct modulation of NF‐κB binding and activity

Metformin is a well‐tolerated drug used extensively in treatment of type 2 diabetes. It reduces NF‐κB nuclear translocation, preventing the activation of the NF‐κB pathway, and has been extensively investigated as a possible SASP modulator (Moiseeva *et al*, 2013). Treatment of Ras‐mutant IRM‐90 fibroblasts with metformin significantly inhibited the secretion of several SASP components, including CXCL‐5, IL‐6, IL‐8 and IL‐1β, by interfering with IKK/NF‐κB activation (Moiseeva *et al*, 2013). In addition, metformin activates AMPKα, resulting in mTOR signalling pathway inhibition (Sinnett‐Smith *et al*, 2013). Resveratrol (Pitozzi *et al*, 2013) and the flavonoids wogonin, kaempferol and apigenin (Lim *et al*, 2015; Perrott *et al*, 2017) are natural compounds thought to interfere with NF‐κB through their interaction with IκB kinases and effectively attenuate the SASP in specific contexts. Glucocorticoids, steroid hormones with potent anti‐inflammatory activity, can also suppress the SASP by modulating NF‐κB transcriptional activity. Treatment with cortisol and corticosterone suppressed the secretion of several SASP components, including IL‐6 and IL‐1α, impairing the ability of the SASP to stimulate breast cancer cell invasion *in vitro* (Laberge *et al*, 2012).

#### Modulation of the upstream regulators of C/EBPβ activity

SASP‐derived pro‐inflammatory effects are linked to JAK/STAT pathway activation, which may sustain cytokine production by activation of the transcription factor C/EBPβ (Faget *et al*, 2019). Treatment of irradiation‐induced senescent adipocytes with the JAK1/2 inhibitor ruxolitinib significantly reduced SASP factor secretion (Xu *et al*, 2015). Ruxolitinib treatment of aged mice notably diminished systemic and adipose tissue inflammation and improved mice fitness, demonstrating anti‐inflammatory potential for treatment of chronic disorders. Additionally, oncogene‐induced senescence is accompanied by dynamic fluctuation of NOTCH1 ligand activity, driving a TGF‐β‐rich secretome while suppressing the pro‐inflammatory SASP through C/EBPβ repression (Hoare *et al*, 2016). NOTCH1 is upregulated within NRAS‐senescent hepatocytes and negatively controls senescence immunosurveillance promoting tumorigenesis. Modulation of this pathway should be considered in potential cancer therapeutic translation.

#### Other ways of modulating the SASP

Other potential SASP‐associated targets have been reported, including the alternative splicing modulator polypyrimidine tract binding protein 1 (PTBP1), which regulates a pro‐inflammatory secretome. Inhibition of PTBP1 attenuated SASP‐induced tumour promotion in a mouse model of hepatocellular carcinoma (Georgilis *et al*, 2018). PTBP1 may therefore be a potential therapeutic target.

Targeting of specific components of the SASP known to be deleterious in certain conditions could provide more precise, potentially biomarker‐directed, therapeutic strategies, including through neutralization of well‐defined cytokines (such as IL‐1, IL‐6 or IL‐8 or their receptors) by monoclonal antibodies. Although such antibodies have been developed (van Rhee *et al*, 2014), their effects on senescence‐associated phenotypes remain uncertain. Intriguingly, canakinumab, a monoclonal antibody directed against IL‐1β and approved for the treatment of cryopyrin‐associated periodic syndrome and other rare autoinflammatory disorders, has been investigated in a number of more common inflammatory disorders—these disorders are linked with senescence and their true mechanism of action may lie in the SASP modulation. Of note, in the phase 3 CANTOS clinical trial for ischaemic heart disease (NCT01327846), patients treated with canakinumab had a very significantly reduced risk of being subsequently diagnosed with lung cancer (Ridker *et al*, 2017). Since pre‐malignant lung adenomas are characterized by accumulation of senescent cells (Collado *et al*, 2005), it is tempting to speculate that senescent cells are targeted specifically by canakinumab.

### Immune activation to target senescent cells

The immune system plays a fundamental role in senescent cell clearance and it is thought that the age‐dependent decline of the immune system is partially responsible for the accumulation of senescent cells in tissues over time. Clearance of senescent cells is driven by CD4(^+^) T cells and monocytes/macrophages through a process known as senescence surveillance (reviewed in Burton & Stolzing, 2018). Natural killer (NK) cells can mediate elimination of senescent hepatic stellate cells in a model of damage‐induced liver fibrosis, resulting in less fibrotic scarring and facilitating fibrosis resolution (Krizhanovsky *et al*, 2008). Accordingly, administration of polyinosinic–polycytidylic acid (polyI:C; a NK Toll‐like receptor 3 [TLR3] ligand) and interferon‐γ enhances the cytotoxic activity of NK cells against activated hepatic stellate cells and ameliorates liver fibrosis (Radaeva *et al*, 2006). This process appears to be mediated by activation of the NK cell receptor NKG2D, which recognizes ligands on the surface of infected, damaged or stressed cells. Ligands of NKG2D are elevated in senescent cells, and NKG2D was required for NK cell‐mediated senescent cell clearance protecting against liver fibrosis (Sagiv *et al*, 2016).

Activation of antibody‐dependent cell‐mediated cytotoxicity (ADCC) may also preferentially eliminate senescent cells. The surface peptidase DPP4 is enriched on senescent human diploid fibroblasts versus normal cells. An anti‐DPP4 antibody triggered ADCC via NK cells, which eliminated senescent cells *in vitro* (Kim *et al*, 2017b). An oxidized form of membrane‐bound vimentin was recently described as a novel marker for senescent murine lung fibroblasts (Frescas *et al*, 2017). The resultant hypothesis that humoral innate immunity may recognize and target the oxidized form of vimentin in senescent fibroblasts is yet to be tested *in vivo*.

A variety of new interventions focused on the activation of the immune system have emerged as effective treatments for a wide variety of diseases. Since senescent cells are immunogenic, reactivation of immune cell effector mechanisms against these cells, or senescent cell manipulation to increase their immunogenicity, are attractive clinical development strategies.

### Bypassing and reverting senescence

Although cell senescence was originally described as an irreversible cell cycle arrest, *in vitro* reactivation of cell proliferation after induction of senescence can be achieved. For example, enforced telomerase activity induces the bypass of replicative senescence without inactivation of the p16^ink4a^/Rb pathway or abrogation of p53 expression (Pellegrini *et al*, 2004). In an OIS model, IL‐6 exerts an antiproliferative effect, and its depletion allows cells to bypass OIS. This phenomenon was associated with the suppression of p15^INK4B^ (Kuilman *et al*, 2008). Inhibition of the chemokine receptor CXCR2 also alleviates replicative senescence and OIS (Acosta *et al*, 2008). Ectopic overexpression of the polycomb group (PcG) protein CBX8, required for proliferation of diploid human and mouse fibroblasts, also allows senescence bypass and immortalizes primary MEFs through direct repression of the *Ink4a‐Arf* locus (Dietrich *et al*, 2007). In a seminal study, *in vivo* reprogramming of somatic cells was shown to restore their proliferative potential (Abad *et al*, 2013), a relevant approach for future applications in tissue repair and regenerative medicine. Interestingly, pools of cells enriched in senescent cells from centenarians were reprogrammed *in vitro* to pluripotent stem cells (Lapasset *et al*, 2011).

These studies open up the possibility of bypassing senescence therapeutically. However, such approaches should be considered with caution. A recent study showed that therapy‐induced senescent (TIS) cells can acquire both functional and phenotypic stemness features in *in vivo* models of lymphoma and leukaemia, developing stronger and more aggressive tumour growth potentials (Milanovic *et al*, 2018). In this pioneering work, lymphoma cells that escaped a previously senescent state presented a higher tumour‐initiating capacity than cells that were never senescent, and this feature was driven by the activation of the canonical Wnt signalling pathway in TIS. This experimental approach used mice carrying loss‐of‐function alleles at the *Suv39h1* locus, and it remains to be seen if the required epigenetic changes associated with reversion of cellular senescence occur in nature.

## Probes for diagnosis

**Figure 4:**
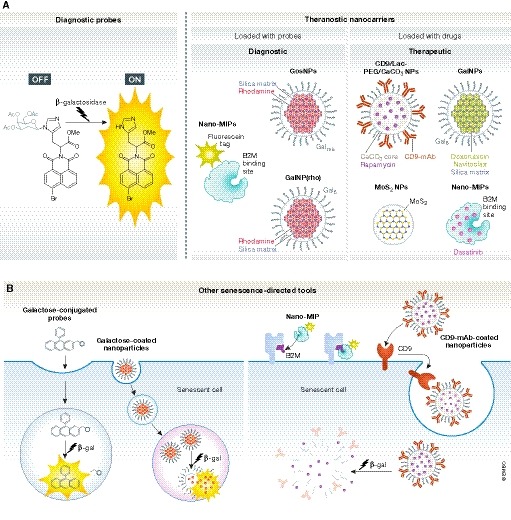
Novel diagnostic and therapeutic approaches for targeting senescent cells: probes and nanoparticles (A) Representative structural images of some of the novel tools developed for the detection and targeting of senescent cells. Diagnostic probes are either fluorescent or chromogenic and can be detected upon β‐galactosidase catalytic reaction. Nanocarriers are loaded or tagged with either fluorescent particles (such as rhodamine) or drugs/senolytics (doxorubicin, navitoclax, rapamycin) for different clinical interventions. Most of the senescence‐directed nanoparticles are coated or conjugated to galactose‐derived residues or have been designed to bind to specific receptors. (B) Tracking the β‐galactosidase activity of senescent cells is one of the commonest strategies for the development of probes and nanoparticles. The enzymatic activity cleaves galactose residues conjugated to endocytosed probes or nanoparticles and allows the release of carriers or the emission of colour/fluorescence within the lysosomal compartment. Other developed tools can bind to receptors present on the membrane to either allow the detection of senescent cells (Nano‐MIPs) or subsequently become endocytosed and processed by β‐galactosidase activity (CD9‐mAb‐coated nanoparticles). B2M, beta‐2 microglobulin; β‐gal, β‐galactosidase; GalNPs, 6‐mer galacto‐oligosaccharides‐conjugated nanoparticles; GosNPs, galacto‐oligosaccharides‐conjugated nanoparticles; MIPs, molecularly imprinted particles; NPs, nanoparticles; PEG, polyethylene glycol.

Senescent cells have increased lysosomal content, with high levels of lysosomal β‐galactosidase, a feature used for decades to highlight senescent cells *in vitro* and *in vivo* (Sharpless & Sherr, 2015). Multiple fluorescent probes for tracking β‐galactosidase activity have been developed over the last years. Although having the potential to target senescent cells, many probes have been validated only in human cells transfected with plasmids harbouring the *Escherichia coli lacZ* gene, resulting in cytoplasmic overexpression of bacterial β‐galactosidase. However, this approach does not recapitulate cellular senescence, as it does not correspond with the endogenous lysosomal β‐galactosidase activity associated with senescence. Also, some probes have been tested in particular human cancer cell lines naturally expressing high levels of lysosomal β‐galactosidase (Table EV3). Here, we focus on fluorescent probes validated in *bona fide* senescent cells/tissues. We believe these sets of probes to be the most promising for translation to *in vivo* models of senescence‐related disorders and/or the accumulation of senescent cells during ageing (see Table EV3 and Fig 4).

### Fluorescent probes validated *in vitro*

The first reported ratiometric two‐photon fluorescent probe to track senescent cells *in vitro*, initially tested in human diploid fibroblasts (HDFs) undergoing replicative senescence, consists of a naphthalene‐based fluorescent moiety (SG1) containing a *β‐D*‐galactopyranoside‐derived benzyl carbamate at the β‐gal hydrolytic site and a solubilizing group (Lee *et al*, 2014). This probe produces a blue‐to‐yellow emission response to β‐gal and has high insensitivity to pH and reactive oxygen species, high photostability and low cytotoxicity. Gal‐Pro, another senescence‐specific fluorescent probe, was recently validated *in vitro* using HDFs undergoing oxidative stress‐induced senescence (Zhang *et al*, 2017). Gal‐Pro is based on a hemicyanine skeleton conjugated with a *D*‐galactose residue via a glycosidic bond displaying near‐infrared emission. The probe exhibited a rapid and sensitive turn‐on response to SAβgal in living cells, with high photostability and low background fluorescence.

### Fluorescent probes validated *in vivo*

The fluorescent probe AHGa can trace senescent cells *in vivo* in mice bearing human melanoma SK‐MEL‐103 tumour xenografts treated with senescence‐inducing chemotherapy (Lozano‐Torres *et al*, 2017). AGHa is an OFF‐ON two‐photon fluorescent probe comprising a naphthalimide fluorophore, an *L*‐histidine methyl ester linker and an acetylated galactose bonded to one of the aromatic nitrogen atoms of the *L*‐histidine through an *N*‐glycosidic bond that can be hydrolysed by β‐galactosidase activity. Whereas tumours of mice treated with palbociclib (a potent senescence‐inducing CDK4/6 inhibitor) and injected intravenously with AHGa displayed a clear fluorescent signal, tumours of untreated mice showed negligible fluorescence, and the probe was not activated significantly in endogenous tissues. The combination of selectivity, sensitivity and straightforward synthesis makes AHGa an efficient and attractive OFF‐ON two‐photon probe for the *in vitro* and *in vivo* imaging of senescence. NIR‐BG is an activatable molecular probe synthesized by glycosylation of a hemicyanine dye and a protected galactosyl bromide, with far‐red excitation, near‐infrared emission and high turn‐on ratio upon SAβgal activation (Wang *et al*, 2019). This probe was validated in a variety of human cancer cells (HeLa and MCF7) undergoing camptothecin and/or radiation‐induced senescence, and also in mice bearing HeLa xenografted tumours undergoing chemotherapy‐induced senescence. HMRef‐βGal is a highly membrane permeable and β‐gal‐sensitive fluorescence probe, which utility was demonstrated for *in vivo* visualization of small tumours initiated by intraperitoneal injection of different ovarian cancer cells expressing endogenous high levels of lysosomal β‐gal (Asanuma *et al*, 2015). HMRef‐βGal remains untested in senescence itself. HMRef‐βGal is based on a spirocyclization strategy that uses a hydroxymethyl rhodol derivative bearing a β‐gal moiety.

Development of fluorescent probes to track β‐gal activity thus represents an innovative approach to monitor senescent cells *in vivo* for potential bioimaging applications. However, clinical applications of fluorescent probes are still challenged by a limited tissue penetration depth (~1 cm), which makes them more suitable for specific tissues, such as the skin. We await next‐generation probes for senescence monitoring, which may include activatable contrast agents (for MRI detection) and radiotracers (for PET detection). Galacto‐conjugation of PET radionuclides could then be an interesting approach to track senescent tissues *in vivo*.

## Nanoparticles as diagnostic and therapeutic tools

Nanomedicine is an innovative approach for cell type‐/biomarker‐/phenotype‐specific cargo delivery. Although the technological success achieved in this field is considerable, incomplete knowledge of nano‐bio interactions and nanoparticle biodistribution in mammals, potential toxic effects, clearance pathways and challenges with scaling up the manufacturing process have impeded the translational applications and commercial development of various promising formulations. Nevertheless, their specificity in cell targeting and versatility in cargo encapsulation makes them ideally suited for modulation/elimination of senescent cells (Table EV4 and Fig 4).

The first targeted cargo delivery system for senescent cells was based on functionalized mesoporous silica nanoparticles (NPs) (Agostini *et al*, 2012). Here, spherical particles (~100 nm size) encapsulating rhodamine were coated with galacto‐oligosaccharides of different lengths (Gos), preventing the release of the cargo out of a silica matrix known as MCM‐41. Cellular uptake of GosNPs occurs via endocytosis, and, after fusion with lysosomal vesicles, the beads are eventually released by exocytosis. Preferential release of rhodamine in SAβgal‐positive human senescent fibroblasts from dyskeratosis congenita patients was observed, but not in (control) proliferative human NSCLC cells.

This silica bead‐based nanotechnology was recently refined by using a homogenous coating consisting of a 6‐mer galacto‐oligosaccharide (Gal) and validated in models of damage‐induced and chemotherapy‐induced senescence (Muñoz‐Espín *et al*, 2018). It was reported that gal‐encapsulated rhodamine, GalNP(rho), is preferentially released in a variety of human cancer cell lines (including melanoma, head and neck squamous cell carcinoma and NSCLC cells) undergoing palbociclib‐induced senescence. GalNP(rho) was selectively activated in senescent lesions *in vivo*, using palbociclib‐treated tumour xenografts and fibrotic lungs damaged by bleomycin. In the latter case, lung epithelial cells and fibroblasts exhibiting enriched signatures of senescence label with rhodamine preferentially upon GalNP(rho) administration when compared with control mice.

In addition to cell labels, nanoparticles can encapsulate small molecules capable of killing senescent cells, potentially widening the therapeutic window of these agents (Table EV4 and Fig 4). Gal‐encapsulated doxorubicin, GalNP(dox), induced apoptosis in palbociclib‐induced senescent cells, but not in control (proliferative) cells, thus validating its therapeutic senescence‐targeting potential (Muñoz‐Espín *et al*, 2018). Of note, GalNP(dox) treatment restored lung function in mice with bleomycin‐induced pulmonary fibrosis and reduced the lung fibrotic scar. In addition, GalNP(dox) promoted tumour regression in combination with palbociclib in mice bearing tumour xenografts of human squamous cell carcinoma or melanoma cells. Besides chemotherapy drugs, encapsulation of senolytics should increase their therapeutic specificity. A combination of palbociclib and galacto‐encapsulated navitoclax also reduced tumour xenograft growth, reinforcing the concept that this nanotechnology is an efficient therapeutic tool to specifically deliver drugs into senescent cells. Reassuringly, galacto‐encapsulation diminished undesirable toxicities of doxorubicin and navitoclax (cardiotoxicity and thrombocytopenia, respectively). Collectively, studies with GalNP nanocarriers provide an important and versatile advance in the ability to deliver small compounds to multiple types of senescent lesions *in vivo*, providing proof of concept of potential therapeutic and diagnostic applications in the clinic.

Porous calcium carbonate nanoparticles (CaCO_3_, ~130 nm size) loaded with rapamycin have also been used to target senescent cells (Thapa *et al*, 2017). CaCO_3_(Rapa) NPs were wrapped with a conjugate of lactose (Lac; to facilitate cargo release by lysosomal β‐galactosidase activity), and polyethylene glycol (to stabilize carriers in blood and prevent opsonization). Lac/CaCO_3_(Rapa) NPs were functionalized with a monoclonal antibody against CD9, a cell surface glycoprotein receptor overexpressed in senescent cells, to further promote senescent HDF targeting. Treatment of HDFs with CD9‐Lac/CaCO_3_(Rapa) resulted in anti‐senescence effects as defined by decreased β‐gal and p53/p21/CD9/cyclin D1 expression, reduced population doubling time, enhanced cell proliferation/migration, reduced expression the SASP components and prevention of cell cycle arrest. Calcium carbonate nanoparticles loaded with a coumarin fluorescent dye, CD9‐Lac/CaCO_3_(C_9_H_6_O_2_), exhibited high cellular uptake by senescent HDFs, indicating that this nanotechnology is also suitable for imaging senescence. In another study, pretreatment of human aortic endothelial cells with molybdenum disulphide nanoparticles (MoS_2_ NPs) inhibited H_2_O_2_‐induced senescence by preventing lysosomal and mitochondrial dysfunction (Ke *et al*, 2018). Exposure to MoS_2_ NPs promoted autophagy in these cells, resulting in improved endothelial cell functionality. Interestingly, another recent study shows that molecularly imprinted nanoparticles (nanoMIPs) can be designed to target epitopes of surface proteins in senescent cells, such as β2 microglobulin (B2M) in senescent EJ bladder cells (Ekpenyong‐Akiba *et al*, 2019). NanoMIPs were internalized after B2M‐binding and had a cytotoxic effect when loaded with the senolytic dasatinib. Fluorescently tagged nanoMIPs detected senescent cells in the abdominal cavity of naturally aged mice, while no signs of toxicity were found at a single dose.

Nanoparticles therefore offer a versatile and novel strategy for *in vitro* and *in vivo* targeting of senescent cells with potential diagnostic and therapeutic applications, including as theranostic tools, aimed at simultaneously detecting and eradicating senescent lesions associated with age and numerous human pathologies.

## Targeting senescence clinically in age‐related disorders

Elimination of senescent cells can ameliorate and even reverse a variety of age‐related disorders in preclinical studies (Muñoz‐Espín & Serrano, 2014; Childs *et al*, 2017; Soto‐Gamez & Demaria, 2017; Ovadya & Krizhanovsky, 2018), holding exciting promises for the development of novel therapeutic strategies against these important pathologies (Fig 1). To this end, clinical trials are in progress where senescent cells are the therapeutic targets of systemically delivered small molecules (Table EV5). Encouraged by successful preclinical results, the combination of D + Q is being tested in some age‐related disorders, including chronic kidney disease, which has multiple systemic consequences (NCT02848131), idiopathic pulmonary fibrosis (NCT02874989) and in haematopoietic stem cell transplant survivors who are at increased risk of premature ageing (NCT02652052).

Most approved cancer therapeutics are designed to eliminate tumour cells by inducing apoptosis. The critical regulator of apoptosis, Bcl‐2 (target of navitoclax), is overexpressed in the majority of small cell lung cancer (SCLC) tumours (Ikegaki *et al*, 1994). Although correlative analyses suggested several putative biomarkers of clinical benefit, navitoclax showed limited efficacy as a single agent in advanced and recurrent SCLC (Rudin *et al*, 2012), suggesting these tumours are not sensitive at steady state and do not have high senescent cell burden, or that sufficient senolytic concentrations were not achieved. Besides promoting apoptosis, many of the standard chemotherapies and targeted therapies used in the clinic can also induce senescence in tumour cells (Ewald *et al*, 2010; Petrova *et al*, 2016), which may be important for at least a portion of therapeutic resistance. TIS translates into slower proliferation rates, but the SASP produced by senescent cells can potentially promote an invasive phenotype and an increased growth in neighbouring non‐senescent tumour cells (Pérez‐Mancera *et al*, 2014; Gonzalez‐Meljem *et al*, 2018). Combining standard senescence‐inducing chemotherapies with senolytic agents therefore represents an attractive approach for treating solid tumours. Several phase 1 and phase 1/2 clinical trials combining senescence‐inducing chemotherapies or targeted therapies with navitoclax are ongoing or completed, including with cisplatin, etoposide and navitoclax in SCLC patients (NCT00878449), dabrafenib, trametinib and navitoclax treating patients with BRAF mutant metastatic melanoma (NCT01989585), and osimertinib and navitoclax in patients with EGFR‐positive advanced NSCLC (NCT02520778). Navitoclax is also combined with gemcitabine (NCT00887757), paclitaxel (NCT00891605), docetaxel (NCT00888108), irinotecan (NCT01009073), erlotinib (NCT01009073), sorafenib (NCT02143401) or trametinib (NCT02079740) in advanced solid tumours. Two trials from the last group have already published results in small subsets of patients with varying solid tumours. A total of forty‐six patients were treated with gemcitabine and a dose escalation of navitoclax (NCT00887757), demonstrating good tolerability and safety for the combination, but no objective responses were observed (Cleary *et al*, 2014). A total of eleven patients received the combination of navitoclax and the tyrosine kinase inhibitor erlotinib (NCT01009073), which was also well tolerated but did not result in any objective responses (Tolcher *et al*, 2015). Another phase 1 study combining navitoclax with carboplatin/paclitaxel in nineteen patients with solid tumours showed modest efficacy (Vlahovic *et al*, 2014).

In these cases, based on heterogeneous cancer‐type patient cohorts, analyses were exploratory, and hence, conclusions on the efficacy should be taken with caution. It is not clear if objective responses were not observed because (i) the tested chemotherapies did not induce senescence in these patients; (ii) senolytics did not achieve sufficient intratumoural concentrations; (iii) senolytics were ineffective at killing senescent cells; (iv) cancer‐type specificities were not representative of preclinical models; or (v) a combination of these/other factors. It is, however, imperative to persist in the approach: future studies should explore promising targeting agents where senescence induction is known to be robust in human patients with specific cancer types well represented by appropriate preclinical models. Embedding translational science into these trial protocols, with the analysis of senolytic effects on pre‐ and post‐treatment biopsies, or the use of senescent cell imaging approaches, should help refine strategies and direct them to patients most likely to benefit.

Age‐related immune senescent remodelling is likely to contribute to the decline of the immune system, chronic inflammatory state, risk for frailty, chronic disease and functional decline in older individuals (Akbar *et al*, 2016). Patients with activated PI3K Delta syndrome present a dominant mutation in the PI3K catalytic subunit p110δ, resulting in T‐cell senescence and immunodeficiency (Lucas *et al*, 2014). Administration of a selective PI3Kδ inhibitor leniolisib (CDZ173) showed a reduction of senescent T cells and a decrease in inflammatory markers in a trial involving six patients (NCT02435173), and an extension study is ongoing (NCT02859727).

Treatment of mice with metformin is associated with a reduction in oxidative stress and inflammation, resulting in extension of lifespan and healthspan (Barzilai *et al*, 2016). Three clinical trials in older patients are evaluating the premise that metformin may be an effective “anti‐ageing” drug (NCT02325245, NCT02570672 and NCT03451006), with changes in frailty indices as their primary outcome measures. A fourth clinical trial (NCT02432287) tests the hypothesis that metformin will result in changes in the transcriptome, reverting the expression profiles of older adults with impaired glucose intolerance towards those seen in younger patients, in muscle and adipose tissue. A phase 3 clinical trial (NCT03309007) aims to investigate the effects of a short treatment with metformin on cellular senescence and autophagy in older adults with pre‐diabetes. Confirming initial hypotheses of improvements seen in autophagy and senescence would justify starting further clinical trials with metformin as an anti‐ageing therapy, but these should ideally be in disease‐specific populations with objective, clinically relevant endpoints.

A phase 2 clinical trial (NCT02874924) is measuring effects of rapamycin (sirolimus) in patients over 70 years old on general parameters of immune health, including levels of inflammatory serum cytokines and polyclonal T‐cell activation. Secondary outcomes include improvement in physical, cognitive and cardiovascular functions. A phase 1 clinical trial (NCT01649960) involving low‐dose rapamycin in older adults with coronary artery disease has already been completed, with the assessment of frailty by physical performance as a primary outcome, and the analysis of the SASP and quantification of the levels of senescent preadipocytes as secondary outcomes. Other approaches are in development, including a further trial (NCT03353597) evaluating the effects of monthly plasma transfusions of young healthy male donors to older subjects (> 40) in order to reverse epigenetic and other markers of senescence. The outcomes of the study are the assessment of cell DNA methylation levels to calculate an epigenetic age, as well as detection of changes in cognitive, renal and pulmonary function, muscle strength, telomere length and expression levels of IGF‐1 and p16^INK4a^ in blood and skin biopsies.

Despite the increasing prevalence and societal burden of age‐related diseases, trials targeting senescence are still relatively few in number, and we await an objective initial proof of principle. It is important that we reassess the best way to translate promising preclinical observations into well‐designed translational approaches and effective therapeutic strategies. Importantly, a number of Biotechnology Companies are now developing translational activities in the field of senescence including, among others: Unity Biotechnology (Bcl‐2 inhibitors), Senolytic Therapeutics (NPs to target SAβgal‐positive cells), Oisin biotechnologies (NPs to target p16‐positive cells), Antoxerene (FOXO4 peptides), CellAge (multigenic senescence signatures), and Everon Biosciences and Siewa Therapeutics (senescence immunotherapy strategies).

## Future perspectives

This review recapitulates a collection of innovative approaches to manipulate and trace cellular senescence, including some already tested and validated in animal models of human pathologies and a number of ongoing studies in humans. Successful clinical translation of strategies targeting senescent cells may have a significant impact on the treatment of multiple human age‐related disorders, as well as increase lifespan and healthspan by delaying the chronological ageing of damaged tissues and organs. Nevertheless, several important challenges must be considered to optimize translation of senescence‐targeted therapeutic strategies to the clinic.

Human cell cultures have provided considerable data on senotherapies (see Tables EV1 and EV4) and senoprobes (see Table EV3). Approaches have employed a variety of senescence‐inducing stressors, different cell types and different regulatory mechanisms. However, most approaches have excluded the complex 3D microenvironment of diseased tissues in living organisms, and their relevance for translation to human diseases remains therefore unclear. The method used to induce senescence (replicative stress, oncogene activation, damaging compounds, irradiation, carcinogens, etc.) strongly determines the key drivers and the signalling pathways involved, a process that is also critically dependent on the cellular type and tissue of origin (Salama *et al*, 2014). Senescent cells in culture are a highly heterogeneous population, and these subpopulations consequently may present different vulnerabilities to senotherapies and senoprobes. It is therefore important that we ask the right clinical question (e.g. Does drug X enhance the efficacy of drug Y in condition Z?) in the right model that is as accurate a representation of the human condition as possible.

Despite major advances, translation of senescence targeting to the clinic should be approached with caution, since senescence plays both beneficial and detrimental roles depending on the pathological context (Muñoz‐Espín & Serrano, 2014) and it is crucial that we examine this thoroughly in appropriate preclinical models. It is well known that cell senescence prevents the expansion of damaged and pre‐malignant cells in a cell autonomous manner, and hence, it is an important barrier against tumorigenesis (Collado & Serrano, 2010). This correlates with the observation that, among other senescence‐related genes, the vast majority of human cancer cells accumulate mutations in the p53‐p21 and/or the p16‐Rb axis (Vergel & Carnero, 2010). A relevant concern is that bypassing or reversing senescence could indeed promote tumorigenesis (Krimpenfort *et al*, 2001). Accordingly, there is evidence that particular populations of therapy‐induced senescent cancer cells can acquire phenotypic and functional stemness features, resulting in cell cycle re‐entry, self‐renewal capacity and a more aggressive tumour phenotype (Milanovic *et al*, 2018). This is less critically important in the context of attempts to improve treatments for patients with advanced cancer where more risk is often ethically acceptable, but nonetheless this requires close interrogation in preclinical models.

Besides cell autonomous roles, the SASP is crucially implicated in the recruitment of T cells and macrophages facilitating immunosurveillance in liver precancerous lesions (Burton & Stolzing, 2018) although the senescent secretome may also be endowed with an intrinsic potential to promote chronic inflammation and tumour progression (Gonzalez‐Meljem *et al*, 2018). It is known that the SASP facilitates tissue repair in disorders caused by severe damage and injury. This is the case for senescent activated stellate cells, capable of limiting liver fibrosis by a reduced secretion of extracellular matrix components, enhanced secretion of extracellular matrix degrading enzymes and increased immunosurveillance (Krizhanovsky *et al*, 2008). In addition, senescent fibroblasts accumulate in granulation tissues of healing cutaneous wounds and express antifibrotic genes (Jun & Lau, 2010). Senescence, however, intriguingly mediates fibrotic pulmonary disease, and removal of senescent cells using senolytics, therapeutic nanoparticles and anti‐inflammatory compounds reverts fibrosis and improves lung function in mice (Hecker *et al*, 2014; Lehmann *et al*, 2017; Schafer *et al*, 2017; Muñoz‐Espín *et al*, 2018). The antagonistic roles of cell senescence in a number of mouse models of human fibrotic diseases highlight the importance of a more detailed understanding of the triggers, intrinsic mechanisms and pathways driving the stable cell cycle arrest and distinct factors secreted by senescent cells before widespread clinical trials, particularly in otherwise healthy populations. Therefore, the development of appropriate and more accurate animal disease models capable of ensuring a causative role of senescence and specific beneficial effects of its manipulation is still a priority before definitively moving into clinical development, with judicious assessment of predicted beneficial/toxic effects of investigational strategies. Moreover, due to significant differences between humans and mice, preclinical studies will require a more detailed characterization of the peculiarities and mechanisms of action of senescence in patients, along with a more extensive validation of senotherapies and senoprobes by using specimens of human tissue *ex vivo* and xenografts in mice (with fully humanized immune systems), to prioritize the most promising disease‐specific candidates for clinical application.

The need to develop specific biomarkers of senescent cells remains a limiting factor for efficient translation of strategies targeting senescence. SASP components and the associated immunoregulation could provide tools for the selection of patients (in a biomarker‐directed manner) and for assessing sensitivity or resistance to senotherapies (and to help drive further clinical development of on‐target, pharmacodynamically active therapeutics). These include screening, diagnostic, monitoring, prognosis and pharmacodynamic tools. In the case of senotherapies, pharmacodynamic biomarkers are keys in measuring the on‐target responses and are crucial for dose optimization studies. Unfortunately, there is currently no universal marker for cellular senescence available (Sharpless & Sherr, 2015), and quantitative methods in basic research have been limited to cytochemical protocols and flow cytometry analyses (Debacq‐Chainiaux *et al*, 2009; Biran *et al*, 2017). One of the first methodologies to image and monitor senescence noninvasively *in vivo* was based on a sequential reporter‐enzyme bioluminescence technology to track β‐gal activity by using Lugal, a caged galactoside‐luciferin conjugate (Wehrman *et al*, 2006). Recent studies validated the use of nanoparticles and fluorescent probes to target senescent cells in mouse models (Lozano‐Torres *et al*, 2017; Muñoz‐Espín *et al*, 2018; Wang *et al*, 2019). Nanoparticles open up the possibility of encapsulating tracers and contrast agents for the imaging of senescence location and burden, and to be used as imaging biomarkers to direct promising therapies to populations most likely to benefit from them. This will however require the adaptation of current preclinical tools to deep tissue penetration bioimaging techniques. For example, diagnostic GalNPs could release gadolinium or positron‐emitting radioisotopes in senescent lesions to allow detection by MRI or PET, respectively. Gal‐encapsulation methods and senoprobes could also serve to monitor the response of solid tumours to the administration of senescence‐inducing chemotherapies, or the senescence burden in patients with senescence‐associated disorders, before and after senotherapy to provide a pharmacodynamic biomarker of response. In addition to cancer and other chronic pathologies, cellular senescence is a defining feature of a wide variety of human pre‐malignant lesions (Collado & Serrano, 2010). Based on this, it is tempting to speculate that GalNPs and senoprobes could be utilized in the early diagnosis of pre‐malignant tumours. Senescence‐specific NPs could also potentially be employed as theranostic tools, aimed at the simultaneous detection and eradication of senescent lesions associated with numerous pathologies, or during ageing. The latter would likely require regular, intermittent interventions to eliminate senescent cells or to manipulate a damaging SASP, while minimizing off‐target effects in normal cells and on‐target manipulation of beneficial senescent cells.

Beneficial senescent cells are plentiful, but biomarkers and drug sensitivities that might distinguish these from pathological senescent cells are lacking. Cellular senescence contributes centrally to the physiological processes of (i) repair, facilitating wound healing through secretion of platelet‐derived growth factor AA (Demaria *et al*, 2014); (ii) regeneration, promoting tissue reprogramming of nearby cells in the context of injury and ageing (Mosteiro *et al*, 2016; Chiche *et al*, 2017; Ritschka *et al*, 2017) including limb regrowth in salamanders (Yun *et al*, 2015); and (iii) embryonic development, playing an active role in tissue remodelling and morphogenesis (Muñoz‐Espín *et al*, 2013; Storer *et al*, 2013). The potential targeting of beneficial senescent cells remains unexplored in the context of senotherapies and senoprobes, not helped by the short lifespans of the widely used animal models. Manipulation of cell senescence during these processes may compromise patient health. In this regard, there are several possible strategies to overcome this problem and optimize selectivity. An important approach will be the development of second generation versions of pharmacologically active compounds and probes already tested in preclinical studies but which target pathological senescent cells specifically, exploiting synthetic lethal vulnerabilities. Another emerging possibility to increase specificity is to modify the therapeutic or diagnostic agent to be activated by an external stimulus or enzymatic reaction. This is the case for galactose‐based nanoparticles, encapsulation methods and probes (Agostini *et al*, 2012; Lozano‐Torres *et al*, 2017; Thapa *et al*, 2017; Muñoz‐Espín *et al*, 2018), which are preferentially activated by the increased lysosomal β‐gal function of senescent cells. Alternatively, accumulation of SASP components and enzymes in the senescent intercellular space might also be employed to stimulate drug delivery systems and inactive pro‐senolytics, or activatable probes, for imaging. Ideally, synthetically lethal compounds would be loaded into nanocarriers targeting senescent (or even pathologically senescent) cells, thereby extensively widening their therapeutic windows. This will require a more detailed information of the diseased cell‐specific signalling pathways and vulnerabilities at the mechanistic level.

In the absence of such data, we should still move forward with the knowledge we have. More refined BCL‐2 family inhibitors capable of preventing dose‐limiting toxicities, such as thrombocytopenia and neutropenia (Cang *et al*, 2015) should be tested. In the case of nanomedicine, translational applications potentially have significant long‐term safety concerns, and it is important to understand the cellular uptake and intracellular trafficking of specific NPs, biocompatibility and biodistribution properties, PK/PD analyses and routes of elimination of the core materials, which heavily rely on their physicochemical properties. For instance, opsonin binding can trigger recognition and clearance by the mononuclear phagocyte system and accumulation of NPs in liver and spleen (Mahmoudi *et al*, 2011), with the potential for sequestration and long‐term toxicity.

Other alternatives might include strategies based on the administration of combinations of senotherapies (such as mTOR inhibitors with other senescence modulating drugs (Han *et al*, 2015), potentially reducing required doses of each agent and off‐target effects), or the use of direct routes of administration to directly target the senescent tissue or organ and reduce exposure of non‐targeted tissues. Examples include inhalation of aerosols for pulmonary drug delivery, or preparations of injectable formulations of senotherapies for *in situ* treatments. Current studies are focusing on the identification of specific epitopes, proteins and surface receptors in senescent cells, which would facilitate the design of antibody‐based therapeutic or diagnostic formulations with increased selectivity and reduced side effects. Some of the identified potential markers, such as DEP1 and B2MG (Althubiti *et al*, 2014), or DCR2 (Collado *et al*, 2005), although overexpressed in senescent cells are also present in other cells and damaged tissues. A possible target is surface DPP4, which is preferentially expressed in senescent but not proliferating human diploid fibroblasts (Kim *et al*, 2017b). A recent *in vitro* study employs CD9 receptors in combination with increased SAβgal activity, for dual NP targeting and drug delivery in senescent cells (Thapa *et al*, 2017), but further studies *in vivo* are required to demonstrate the translational applications of these approaches. Short or intermittent exposures to senotherapies may also decrease their potential of driving adverse effects while maintaining their therapeutic benefits. This might be particularly relevant in treatments aimed at maintaining tissue homeostasis and function, and to delay ageing. However, it remains unclear how to justify preventative senotherapies outside the context of disease. Extensive preclinical testing will have to be completed before moving clinical trials towards otherwise healthy populations.

Besides the aforementioned strategies to manipulate cell senescence, other emerging features of senescent cells may be used to develop novel therapeutics, biomarkers and/or diagnostic tools in the near future. Senescent cells and SASP factors are accompanied by a significantly increased release of extracellular vesicles and exosomes (Lehmann *et al*, 2008) containing proteins, lipids and microRNAs, affecting nearby tissue and potentially having relevant roles in immune regulation (Xu & Tahara, 2013; Urbanelli *et al*, 2016; Borghesan *et al*, 2019). Small exosome‐like extracellular vesicles can be important mediators of the pro‐tumorigenic functions of the senescent secretome. This process involves extracellular vesicle‐associated EphA2 secreted from senescent cells, which binds to ephrin‐A1 that is highly expressed in several cancer cell types and promotes proliferation (Takasugi *et al*, 2017). Exosomes released by senescent prostate cancer cells subjected to radiation therapy were enriched in B7‐H3 protein, an immune checkpoint ligand. It is tempting to speculate that manipulation and identification of extracellular vesicles secreted by senescent cells might be used in (immuno)therapeutic approaches. Another interesting alternative to modulate the SASP and the immune response could be through modulation of the cGAS‐cGAMP‐STING signalling pathway. This pathway detects cytoplasmic chromatin fragments in response to DNA damage in senescent cells, activates type I interferons and other cytokines, and mediates autoinflammatory diseases (Li & Chen, 2018). Cyclic guanosine monophosphate (GMP)–adenosine monophosphate (AMP) synthase (cGAS) and the adaptor protein STING are key drivers of the senescent secretome in primary human cells and in mice (Dou *et al*, 2017; Glück *et al*, 2017; Yang *et al*, 2017). Mice deficient in cGAS and STING show impaired immunosurveillance of oncogenic RAS and reduced tissue inflammation after ionizing radiation. Furthermore, this pathway is activated in cancer cells and correlates with pro‐inflammatory gene expression in human cancers (Li & Chen, 2018). It is therefore reasonable to explore novel therapeutic and/or diagnostic strategies based on the manipulation of the cGAS‐cGAMP‐STING signalling pathway. Several small‐molecule antagonists of STING with efficacy in the treatment of autoinflammatory disease in mice have already been characterized (Haag *et al*, 2018).

The wealth of potential therapeutic targets that senescence presents in disease are myriad. However, our study of the roles and pathways of senescence in specific pathologies is much less advanced. We advocate translational studies to elucidate these in the human disease of interest, with appropriate preclinical validation of putative therapeutic strategies in appropriate models before the strategies predicted to be most safe and efficacious are considered for clinical trials.

## Concluding remarks

Global populations continue to age, increasing the prevalence of chronic age‐related pathologies, and producing an associated public health epidemic. Senescent cells accumulate during ageing, causing tissue dysfunction, and are associated with a wide variety of age‐related disorders, both in humans and animal models. Preclinical studies have convincingly concluded that the elimination of senescent cells can ameliorate and even revert the pathological manifestations of multiple disorders in mice (Fig 1). Many challenges must be overcome to ensure successful translational application of strategies. These include the need for: (i) a better understanding of the triggers and signalling pathways driving the senescent arrest and the different secretomes in the context of a particular disease; (ii) the development of more advanced and clinically relevant animal models capable of distinguishing on‐target from off‐target effects for each condition (Kirkland & Tchkonia, 2015); (iii) an increase in the selectivity of senotherapies or senoprobes, to reduce known and potential toxicities; (iv) solid experimental data concerning the biodistribution properties, PK/PD modelling and routes of elimination of nanocarriers; and (v) the optimization of the therapeutic/diagnostic window of free/encapsulated senolytic agents/combinations. Owing to the heterogeneity of patients and the complexity and multifactorial nature of ageing and age‐related disorders, it is likely that future interventions against cellular senescence will be biomarker‐ and context‐dependent (personalized), where the risk–benefit ratio can be more clearly understood. While surprisingly few early‐phase clinical studies on senotherapies have been initiated, we expect this to balloon in coming years and hope that these are designed prudently. We are entering an exciting era, where we will be able to move anti‐senescent therapies towards medical applications, a strategy that may have important impacts on precision medicine, healing, tissue repair, regeneration and, ultimately, on human longevity.

**Pending issues**

(i) A more detailed knowledge of the cell‐specific triggers and signalling pathways driving different senescent programmes and secretomes and how they correlate with distinct age‐related disorders. In particular, by analysing human samples and not only by the use of animal tissues.
(ii) Identification of specific cell membrane/intracellular/extracellular markers/targets of senescent cells, in order to develop next‐generation senolytics, senoprobes or nanoparticles with increased selectivity and reduced off‐target effects.
(iii) Development of more clinically relevant animal models recapitulating age‐related human diseases.
(iv) Optimization of therapeutic/diagnostic doses of agents targeting senescence, with preclinical examination of biodistribution, PK/PD, toxicity and safety aspects in animal models.
(v) Evaluation of the first human early‐phase clinical trials to provide proof of principle of the pharmaceutical impact of senotherapies on age‐related disorders and ageing.

### Conflict of interest

The authors declare that they have no conflict of interest.

### For more information

(i) https://clinicaltrials.gov

## Supporting information



Table EV1

Table EV2

Table EV3

Table EV4

Table EV5
